# Supplementary material for: The genome sequence of a small dung beetle,  Volinus sticticus (Panzer, 1798), formerly known as Aphodius sticticus
Source: Wellcome Open Res. 2025 Apr 18;10:195. [Version 1] doi: 10.12688/wellcomeopenres.23692.1 (PMC12149814; doi:10.12688/wellcomeopenres.23692.1)
Supplement: Peer review history

# Reviewer report: Reviewer response for version 1

**DOI:** 10.21956/wellcomeopenres.26138.r124025

**Published:** 2025-06-09

**Authors:** Doga Cedden

**Affiliations:** 1 University of Göttingen, Göttingen, France

The authors report a high-quality chromosome-level genome assembly from the small dung beetle, *Aphodius sticticus. *The background regarding the beetle is sufficient. The authors provide a thorough quality control of the assembly. The genome assembly and generated sequence data are publicly available. 

 -Please consider providing a scale in Fig. 1.

 I have no further comments.

Are sufficient details of methods and materials provided to allow replication by others?

Yes

Is the rationale for creating the dataset(s) clearly described?

Yes

Are the datasets clearly presented in a useable and accessible format?

Yes

Are the protocols appropriate and is the work technically sound?

Yes

Reviewer Expertise:

I work on coleopteran pests usign RNA interference and RNA-seq

I confirm that I have read this submission and believe that I have an appropriate level of expertise to confirm that it is of an acceptable scientific standard.

# Reviewer report: Reviewer response for version 1

**DOI:** 10.21956/wellcomeopenres.26138.r124019

**Published:** 2025-06-09

**Authors:** Lindsey C Perkin

**Affiliations:** 1 United States Department of Agriculture ARS, College Station, Texas, USA

The manuscript presents a nice genome for the small dung beetle, Volinus stricticus. The authors follow the community standard for collecting, sequencing and assembling reference quality genomes.

 The protocols and methods are well defined and easy to follow. The data is supported and summarized by many tables and figures.

 The datasets are referenced and in an accessible format.

Are sufficient details of methods and materials provided to allow replication by others?

Yes

Is the rationale for creating the dataset(s) clearly described?

Yes

Are the datasets clearly presented in a useable and accessible format?

Yes

Are the protocols appropriate and is the work technically sound?

Yes

Reviewer Expertise:

agriculturally significant pests of row crops

I confirm that I have read this submission and believe that I have an appropriate level of expertise to confirm that it is of an acceptable scientific standard.

# Reviewer report: Reviewer response for version 1

**DOI:** 10.21956/wellcomeopenres.26138.r124018

**Published:** 2025-05-30

**Authors:** Hume Douglas

**Affiliations:** 1 Agriculture and Agri-Food Canada, Ottawa, Canada

This article efficiently presents the information that genome users will need. This article is part of a series, and all are well written.

 The information about the beetle is sufficient. The species is a good choice for sequencing. 

 The methods are well presented.

 The results are well presented.

 The sequence data, mapping and chromosome information will all be appreciated by researchers.

Are sufficient details of methods and materials provided to allow replication by others?

Yes

Is the rationale for creating the dataset(s) clearly described?

Yes

Are the datasets clearly presented in a useable and accessible format?

Yes

Are the protocols appropriate and is the work technically sound?

Yes

Reviewer Expertise:

Beetle systematics

I confirm that I have read this submission and believe that I have an appropriate level of expertise to confirm that it is of an acceptable scientific standard.
